# Response to neoadjuvant chemotherapy in breast cancer: do microRNAs matter?

**DOI:** 10.1007/s12672-022-00507-z

**Published:** 2022-06-07

**Authors:** Dinara Ryspayeva, Volodymyr Halytskiy, Nazarii Kobyliak, Iryna Dosenko, Artem Fedosov, Mariia Inomistova, Tetyana Drevytska, Vitalyi Gurianov, Oksana Sulaieva

**Affiliations:** 1 Department of Oncohematology and Adjuvant Treatment Methods, National Cancer Institute, Lomonosova str, 33/43, Kyiv, 03022 Ukraine; 2 Palladin Institute of Biochemistry of the National Academy of Sciences of Ukraine, Kyiv, 01054 Ukraine; 3 Institute of Molecular Biology and Genetics of the National Academy of Sciences of Ukraine, Kyiv, 03143 Ukraine; 4 Endocrinology Department, Bogomolets National Medical University, Kyiv, 01601 Ukraine; 5 Medical Laboratory CSD, Kyiv, 03148 Ukraine; 6 Bogomolets Institute of Physiology of the National Academy of Sciences of Ukraine, Kyiv, 01024 Ukraine; 7 Sumy State University, Sumy, Ukraine

**Keywords:** Resectable breast cancer, Neoadjuvant chemotherapy, Response to therapy, microRNA, miR-124, miR-137, miR-34a, miR-155, miR-373

## Abstract

**Background:**

Conventionally, breast cancer (BC) prognosis and prediction of response to therapy are based on TNM staging, histological and molecular subtype, as well as genetic alterations. The role of various epigenetic factors has been elucidated in carcinogenesis. However, it is still unknown to what extent miRNAs affect the response to neoadjuvant chemotherapy (NACT). This pilot study is focused on evaluating the role of miR-34a, miR-124a, miR-155, miR-137 and miR-373 in response to NACT.

**Methods:**

That was a prospective study enrolling 34 patients with histologically confirmed BC of II-III stages. The median age of patients was 53 (47–59.8) years old, 70.6% of whom were HR-positive. MiRs levels were measured in the primary tumor before and after NACT. The response to therapy was assessed after surgery using the Miller-Payne scoring system. To establish the role of miRs in modulating response to NACT the Cox model was applied for analysis.

**Results:**

BC demonstrated a great variability of miRs expression before and after NACT with no strong links to tumor stage and molecular subtype. Only miR-124a and miR-373 demonstrated differential expression between malignant and normal breast tissues before and after therapy though these distinctions did not impact response to NACT. Besides miR-124a and miR-137 levels after NACT were found to be dependent on HR status. While miR-124a levels increased (p = 0.021) in the tumor tissue, the expression of miR-137 was downregulated (p = 0.041) after NACT in HR positive BC.

**Conclusions:**

The study revealed differences in miR-124a and miR-373 expression after NACT in primary BC tissues. Although miRs levels did not impact the response to NACT, we found miR-124a and miR-137 levels to be related to hormonal sensitivity of BC.

## Introduction

For decades breast cancer (BC) has been demonstrating the highest incidence among all cancers in women around the world [1]. By now BC prognosis and prediction of response to therapy are based on TNM staging and molecular subtype. Besides the role of various epigenetic factors has been shown in BC. In recent years, microRNAs (miRs) have attracted significant interest due to their regulatory involvement in cancer initiation, progression and metastasis [2–4]. Studies have shown that certain miRs signatures in various cancers exhibit differential expression and correlate with tumor aggressiveness, response to the therapy and patients’ outcome [5–7]. Certain miRs expression level is closely related to the histopathological parameters and molecular subtypes of BC as well as the response to treatment and prognosis [8–13]. It has been shown that some miRs activate the functions of oncogenes while others stimulate tumor suppressors in BC [4]. However, the exact relationship between some miRs and the biological behavior of BC remains unclear and requires further research.

Among many various miRs, some demonstrate tumor-suppressing epigenetic function. The role of miR-34a, miR-137 and miR-124a as antioncomiRs and tumor suppressors was shown in many studies. Downregulation of these miRs was found to be associated with unfavorable prognosis in many cancers, including BC [14]. Low expression of miR-34 was significantly associated with TNM stage and higher grade of BC [15–17]. At the same time, some authors declare that high level of miR-34a impacts BC aggressiveness but not the survival of BC patients [18]. In contrast, Peurala et al. found that activated miR-34a expression independently exerted a lower risk of recurrence or death from breast cancer [16]. Similarly, conflicting data were illuminated concerning miR-137. Both high and low levels of miR-137 were found to be associated with unfavorable prognosis in TNBC [19, 20] and a decreased patient survival [21].

miR-155 and miR-373 have been recognized as oncogenic miRs (oncomiRs). There is a growing body of evidence demonstrating the diagnostic and prognostic significance of miR-155 in BC. Its upregulation was reported as an indicator of breast tumor invasiveness, late-stage BC with lymph node metastases, high grade and poor prognosis [22–24]. However, in another study miR-155 overexpression was determined as protective and correlated with a better BC prognosis [25]. Overexpression of miR-373 was found to be associated with lymph node metastases in BC [26]. The patients with low miR-373 expression had shorter overall (OS) and progression-free survival (PFS) [27]. Alternatively, the suppressive role of miR-373 was demonstrated in reducing tumor cells invasiveness [5, 28, 29].

What makes miR-124, miR-34a, miR-155, miR-137 and miR-373 attractive for further analysis is the fact that these miRs are related with DNA repair, can modulate genomic instability and sensitivity to genotoxic chemotherapeutic agents [30–32]. Although the role of different miRs in BC biology and outcomes is widely discussed, their role in response to chemotherapy is less discovered and more controversial. This defined the goal of the study, focused on establishing the role and possible predictive significance of miR-124a, miR-34a, miR-155, miR-137 and miR-373 in response to neoadjuvant chemotherapy (NACT) in patients with BC.

## Methods

### Study design

A total of 34 patients with histologically confirmed invasive BC were recruited in the study at National Cancer Institute (NCI; Ukraine) in 2016–2017. The study protocol was approved by the local Ethics Committee of NCI (protocol 81, issued by 05.05.2016).

Inclusion criteria were as follows: histologically confirmed invasive BC at core needle biopsy; clinical stages II-III; both NAC and surgical procedures were performed in NCI; patients had not received chemotherapy, radiation, and endocrine therapy before enrollment into the study. Only patients who provided written informed consent on participating in the study were enrolled.

Patients with histologically confirmed distant metastasis at the time of diagnosis and those who had inflammatory carcinoma were excluded from the study. Cases with inappropriate tumor samples according to pathologist reports and/or insufficient RNA for testing were excluded.

The clinical stage of TNM was assessed by physical examination and mammography, ultrasound of the breast, axilla and abdomen, chest X-ray or computed tomography scan. Demographic data and medical history were collected from medical records.

Before treatment, ultrasound-guided core needle biopsies using an automatic biopsy instrument (Fast Gun, Sterylab) were collected. Several samples were obtained from each lesion, half of them were enclosed in Eppendorf tubes containing DNA/RNA Shield reagent for sample preservation, then frozen at − 70 °C for subsequent miRNAs profiling. The rest was provided for histological assessment and further immunohistochemistry.

Patients received NACT intravenously once a day in 21-day cycles. Preoperative treatment was 2–4 cycles. The following NACT regimens were used: FAC (doxorubicin 50 mg/m^2^, cyclophosphamide 500 mg/m^2^, 5-fluorouracil 500 mg/m^2^), AT (doxorubicin 50 mg/m^2^, docetaxel 75 mg/m^2^ or paclitaxel 175 mg/m^2^), AC (doxorubicin 60 mg/m^2^, cyclophosphamide 600 mg/m^2^) and TC regimen (docetaxel 75 mg/m2 plus cyclophosphamide 600 mg/m^2^).

After NACT all patients underwent breast conserving surgery or mastectomy with axillary lymph node dissection. Specimens from tumor and surrounding non-affected by carcinoma tissues were collected as it was described above and frozen at − 70 °C for subsequent miRNAs profiling.

### Histopathological and immunohistochemical assessment

The grossing of the surgical material was performed according to CAP-recommended protocols and followed by further samples processing, paraffin embedding and histological slides staining. Tissues taken by core biopsies and during surgery were examined by 2 independent pathologists. All tumors were graded according to the Nottingham grading system. Tumor size, histological type and grade, a number of positive lymph nodes were evaluated. Pathological response to NACT was assessed using the Miller-Payne scoring system as reported previously [33]. Grades 1 and 2 were categorized as a poor response, and Grades 3–5 were referred to the category of good response on therapy [34]. Besides, pathological complete response was defined as a non-invasive residual disease in breast tissue and axillary lymph nodes after NACT as reported previously [35].

Every case was assessed by immunohistochemical staining for estrogen receptor (ER) (clone EP1; Dako, Denmark), progesterone receptor (PgR) (clone PgR 636; Dako) and Ki-67 (clone MIB-1; Dako). Hormone positivity was defined in case of > 1% cells with nuclear reaction. HER2 status was assessed by immunohistochemical or fluorescent in situ hybridization by CAP-recommendations. According to the results of immunohistochemistry, the five subtypes of breast cancer were identified: Luminal A, Luminal B (HER-), Luminal (HER+), HER2-enriched and triple-negative (TNBC).

### Analysis of miRNA expression

RT-qPCR (reverse transcription quantitative polymerase chain reaction) assays were performed to determine the expression level of miR-124a, miR-137, miR-34a, miR-155 and miR-373. Besides the representative samples of tumor beds were obtained after the surgery.

To isolate total RNA and microRNA, the NucleoSpin miRNA kit (Macherey–Nagel, Germany) was used according to the manufacturer's protocol. The results were detected in real-time using the 7500 Real-Time PCR System amplifier and a mixture of Universal PCR Master Mix reagents (Applied Biosystems, USA). TaqMan® MIRNA Assays primers (mmu-, hsa-miR-155, hsa-miR-34a, hsa-miR-373, mmu-miR-137) were applied to detect microRNAs in the qPCR reaction.

The following temperature regimen was taken for the PCR reaction: activation of AmpliTaq Gold DNA polymerase at 50 °C for 2 min, primary denaturation 95 °C for 10 min, accumulation of the amplification product for 40 cycles 95 °C—15 s, 60 °C—60 s. The Ct method was made to normalize the expression levels of target genes by correcting the difference in the amount of cDNA with regard to the endogenous control for microRNA U6B (TaqMan® MicroRNA Control RNU6B). miRNA expression levels were calculated by the ΔCt method.

### Statistical analysis

MedCalc® Statistical Software v. 20.025 (MedCalc Software Ltd, Ostend, Belgium; https://www.medcalc.org; 2022) was used for the analysis. Statistical data were analyzed in the same patients before and after NACT. To control the results, paired adjacent non-tumor tissues in the postoperative specimens were collected from the same patients.

Pearson test χ^2^ (for qualitative variables) and T–Wilcoxon test for paired samples (for quantitative variables) were applied to comparing clinical and pathological data in the groups before and after treatment. A Mann–Whitney test was used to compare the differences of HR-positive and HR–negative BC. A Kruskal–Wallis’s test and post hoc analysis (Dunn’s test) were performed to compare the differences in miRs levels of more than two groups.

Multivariate analysis of the prognosis factors with a Cox proportional hazards model was performed. p < 0.05 was considered to indicate a statistically significant difference.

## Results

The median age of the patients was 53 years old with an interquartile interval (IQI) of 47–59.8 years. Baseline clinical and pathological data of the patients are represented in Table 1.

**Table 1:** Clinical-pathological characteristics of patients

Characteristics	Number of patients	%
Age	53 (47–59,8)	
Before 55	18	52.4%
After 55	16	47.6%
Hormone receptors		
HR+	24	70.6%
HR−	10	29.4%
Molecular subtype		
Luminal A	6	17.7%
Luminal B	13	38.2%
LumB-HER2+	5	14.7%
HER2 enriched	4	11.7
TNBC	6	17.7%
Nodal status		
Node positive	31	91.2%
Node negative	3	8.8%
Stage		
I	0	0
IIA	4	11.8%
IIB	13	38.2%
IIIA	9	26.5%
IIIB	7	20.6%
IIIC	1	2.9%
MP-score after surgery		
Grade 1	0	0
Grade 2	14	41.2%
Grade 3	16	47.1%
Grade 4	1	2.9%
Grade 5	3	8.8%

### MiRs levels in primary untreated tumor

The levels of miRs demonstrated high variability within breast cancer tissues taken before NACT. We did not find significant differences in each miR level between various molecular subtypes of BC, stages and grades. However, there was a trend in miRs expression in BC depending on hormone sensitivity status. miR124 and miR-155 expression was higher in HR-negative cases, while miR-137 expression prevailed in HR-positive BC though these differences were not statistically significant.

When assessing the relation between miRs and tumor characteristics we found that miR-137 and miR-373 levels significantly differed (p = 0.038 and p = 0.049, correspondingly) in small (T1-2) compared to large carcinomas (T3-4), though there was no relationship between miRs expression and nodal status or prognostic stage.

Interestingly, miR-155 expression was related to the patients’ age demonstrating a higher range in women aged over 55 years as compared to younger patients (p = 0.0403). At the same time, there were no distinctions of other miRs levels with regard to age.

### Assessment of the predictive value of miRs

When assessing the response to therapy we found 3 patients with a complete pathological response. According to the assessment by MP-score, there were 20 patients (58.8%) with good responses and 14 cases (41.2%) with poor responses to therapy. There was no relationship between hormonal receptors status, patients age, tumor characteristics and frequency of the response to NACT among observed patients. Besides, we failed to find the possible prognostic value of assessed miRs (Table 2) in predicting the response to NACT.

**Table 2:** Cox proportional analysis of the prognostic factors

Covariate	Coefficient of Cox model, b	Standard error, SE	p
miR-34a	− 0.088	0.080	0.271
miR-124a	− 48.0	46.6	0.303
miR-137	− 6.8	11.0	0.537
miR-155	− 0.19	0.26	0.465
miR-373	− 0.43	2.89	0.883
Age	− 0.039	0.027	0.147
Stage	0.15	0.72	0.835
T	− 0.04	0.36	0.914
N	− 0.08	0.55	0.891
Ki-67	0.016	0.017	0.337

### miRs levels after therapy

Measurement of miRs levels after NACT in tumor bed revealed the significant upregulation of antioncoMiR-124a expression (p = 0.004) and prominent downregulation of miR-373 expression (p = 0.006). Notably, miRs levels were much higher in the adjacent tissues not affected by the carcinoma as compared to the tumor bed both before and after NACT. The rest of the assessed miRs did not demonstrate significant changes after therapy (Table 3).

**Table 3:** The levels of miRNA from primary tumor, after NACT and adjacent non-tumor tissues

miRNAs	N	Expression level of mRNA Median (25–75 P)			p
		tumor tissue		adjacent non-tumor tissue (n = 15)	
		Before treatment	After NACT		
miR-34a	34	0.814 0.296–2.144	0.688 0.144–4.000	0.933 0.435–2.841	0.733^a^
miR-124a	32	0.00325* 0.000623–0.0170	0.0192** 0.00337–0.107	0.149 0.0266–0.616	0.001* 0.004**
miR-137	29	0.0012 0.000206–0.0204	0.000213 0.0000396–0.00176	0.00052 0.0000256–0.00419	0.146^a^
miR-155	34	0.789 0.177–5.657	0.407 0.0884–7.464	0.707 0.278–2.327	0.741^a^
miR-373	29	0.000488 0.0000405–0.00427	0.0000678* 0.00000915–0.00112	0.00378 0.00159–0.0110	0.006*

^*^The difference from the group with non-tumor tissue, p < 0.05 (post hoc analysis Dunn’s test)

^**^The difference from the group before treatment, p < 0.05 (Wilcoxon test for paired samples)

^a^Significance levels of differences by Kruskal–Wallis test

**Fig. 1:**
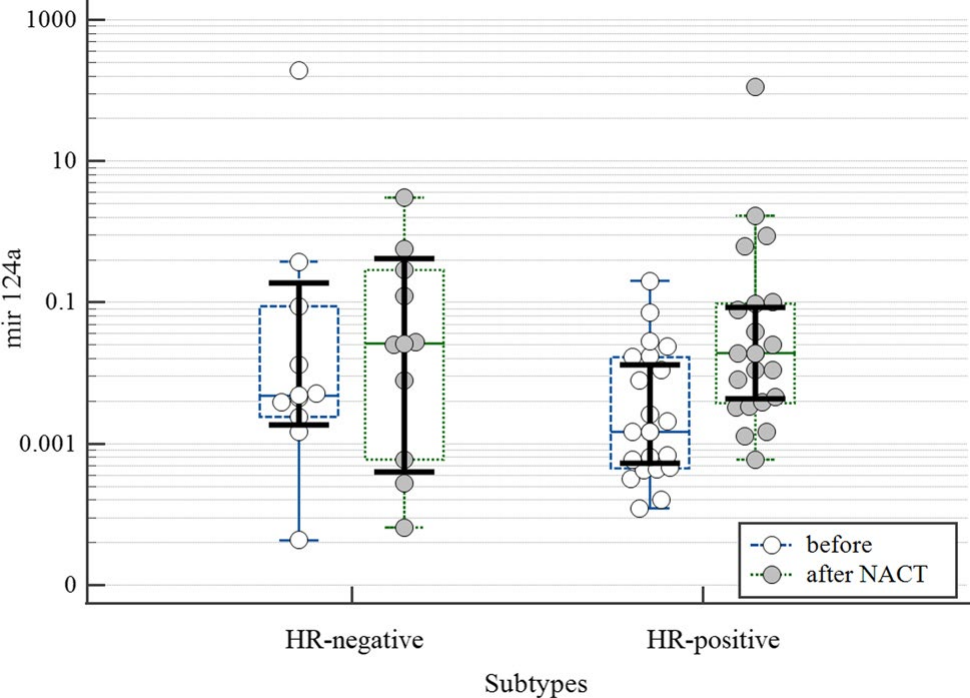
The increased expression of miR-124a in primary tumor tissue after treatment in patients with HR-positive BC (p < 0.05)

**Fig. 2:**
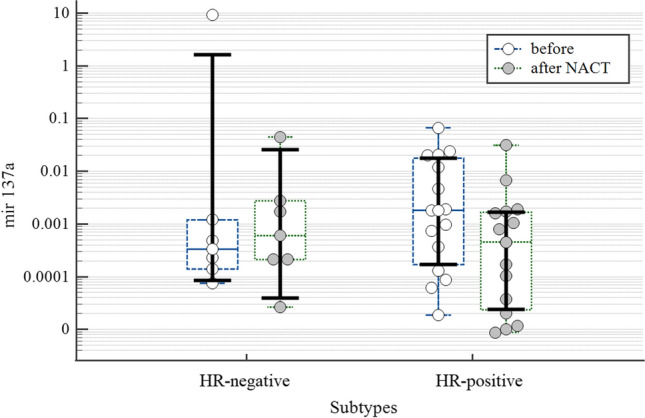
The expression of miR-137 in primary cancer tissues differed after treatment of patients with HR-positive BC (p < 0.05)

Subgroup analysis revealed a difference of two suppressor miRNAs in HR-positive BC patients. The dynamics of the expression levels of tumor-suppressor miR-124a and miR-137 in HR-positive BC is shown in the figures (Fig. 1, Fig. 2). While the expression of miR-124a in the tumor tissue significantly increased after the treatment (p = 0.021) the expression of miR-137 was downregulated (p = 0.041) thought it did not differ from the levels of non-tumor tissue (p = 0.146) (Table 3). At the same time, the differences in the dynamics of expression of the studied miRNAs in the tumor tissue of patients with HR-negative BC were not found.

Thus, NACT induced upregulation of antioncomiR-124a and downregulation of miR-373 expression mostly. However, there was no relationship between these miRs levels and response to therapy. BC of different hormonal sensitivity differed in miR 124a and miR137 expression in response to NACT.

## Discussion

Although we have not revealed the predictive significance of miRs in defining the response to NACT in BC, our study uncovered at least two important facts: first, there were significant changes in miR-124 and miR-373 after NACT compared to the initial levels; second, miRs levels were related to the hormonal status of BC.

The significant downregulation of miR-373 was found in BC patients after NACT. It is of note that, miR-373 was first identified as a human embryonic stem cell-specific miRNA, modulating cell proliferation, apoptosis, senescence, migration and invasion, reaction to hypoxia and DNA damage repair response [36]. This miR yields diverse targets and various functions [5]. miR-372/373, a cluster of stem cell-specific microRNAs transactivated by the Wnt pathway, has been reported to be dysregulated in various cancers [37–39]. In colorectal cancer cells overexpression of miR-373 was associated with elevation of stemness‐related pathways, including Hedgehog, c‐Myc and Nanog signaling [37]. Besides it was reported that miR-373 can enhance the levels of the mesenchymal markers, including β-catenin, N-cadherin and vimentin, and decrease the level of the epithelial markers, E-cadherin and claudin, contributing to BC enhanced cells invasiveness and metastasis development [40]. Overexpression of miR373 was associated with hyperactivation Wnt/*β*-catenin signaling was hyperactivated in cancer cells, facilitating the epithelial-mesenchymal transition (EMT). Such an effect of miR-373 is explained by direct suppression of Dickkopf-1 (DKK1), a negative regulator of the Wnt/*β*-catenin signaling cascade [41]. So that upregulation of miR-373 is associated with cancer cells dedifferentiation and enhanced invasiveness. Besides, miR-373 overexpression contributes to hypoxia-induced downregulation of RAD52 and RAD23B, the components of NER and HRR machineries respectively [42]. This can affect both the mechanisms of genomic instability and sensitivity to chemotherapeutic agents.

Alternatively, the levels of miR-124 increased after NACT as compared to the levels in biopsy though miR-124 levels did not reach the concentration in the non-affected adjacent tissues. miR-124 known as oncosuppressor was shown to be involved in the inhibition of cancer cells proliferation, limiting tumor growth [43]. At the same time, Chen et al., demonstrated miR124 contribution in increasing the sensitivity of cells to chemotherapy in vitro [44, 45]. Assessment of miR-124 levels with respect to hormonal sensitivity of BC revealed that enhancement was more prominent in HR-positive cases as compared to HR-negative tumors. Previous studies demonstrated the relationship between estrogen receptors and miR-124 expression [46]. Authors showed that the silence of ERα significantly inhibited miR-124 expression. It is also worth noting that enhancement of miR-124 expression inhibits cell proliferation, migration and invasion in ER-positive BC cells [46]. On the other side, miR-124 expression is considered to be an independent marker of a favorable prognosis in many oncological diseases including invasive BC [44, 47], while reduced miR-124a expression correlated with lymph node metastases and low OS [48, 49]. The relationship between miR-124 and cancer cells behavior could be realized through inhibition of AKT2—the well-known oncogene and the direct target of miR-124. At the same time increased activation of AKT2 through different regulatory pathways (including estradiol) can attenuate miR124 expression in BC [46]. Importantly, miR-124 is involved in hypoxia-induced factor-1 (HIF-1) signaling pathway through targeting STAT3 [50]. By this way miR-124 is responsible for reversing the resistance to doxorubicin in breast cancer stem cells. It was also demonstrated that miR-124 regulates translation of several DNA-repair–related genes, including ATM interactor (ATMIN) and poly (ADP-ribose) polymerase 1 (PARP1). Although we did not find the significant predictive value of miR-124 expression among observed cases, the role of miR-124 yields high translational potential [50]. Targeting miR expression seems to be relevant for modulating DNA damage response and overcoming drug resistance in cancer.

This study has also illuminated the alternative changes in miR-137 levels with the reduction in HR+ carcinomas after NACT. It is considered that miR-137 negatively regulates a wide range of downstream targets in various types of cancer and can target multiple transcripts [51, 52]. Namely, miR-137 negatively regulates the orphan nuclear receptor estrogen-related receptor α (ERRα) and impairs cell proliferation and migration in BC cells [52]. In addition, miR-137 can suppress the growth and migration of HR+ BC cells at least partly through cell cycle proteins cyclinE1 and WNT11 which are the elements of downstream of ERRα [52]. Wnt11 is known to be activated during embryogenesis and it is upregulated in a wide range of malignancies and especially in metastatic disease [39, 53]. WNT11 was also shown to mediate WNT/PCP signaling via the RHO/ROCK pathway affecting the aggressive phenotype of breast cancer cells [54]. As a contributor to TGF-β signaling pathways, Wnt11 also impacts EMT, affecting cancer cell migration and invasion [55, 56]. So the reduction of miR-137 can attenuate BC aggressiveness. It is challenging to explain alternative changes in the dynamics of miR-124 and miR-137 after NACT in HR+ BC. It is obvious that particular molecular subtypes of BC differ from each other due to distinctions in signaling and proportions of cancer stem cells [19]. Several factors might impact the differential relations between miRs, biological characteristics of BC and response to therapy. First, estrogens can directly modify the DNA damage response and DNA repair mechanisms, modulating by this way the mechanisms of chemosensitivity to genotoxic agents [57]. On the other hand, alteration of DNA repair can affect estrogen signaling pathways, modulating BC molecular subtype and chemoresistance [58]. Finally, lack of HR-sensitivity can affect the stemness of BC, that also impacts the response to therapy. miRs regulatory network is quite complex and the differential regulatory effects of miRNA cannot be explained by differences in expression alone. The effects of some miRs clusters can be multiple, affecting common and alternative targets involved in the coordination of different biological processes [59]. Perhaps, further assessment of both miRs and their targets in BC before and after NACT might shed light on the mechanisms defining the response to therapy.

Limitations of the study. That was a pilot study with a limited sample. Taking into consideration the heterogeneity of the BC and high variability of miRNA expression, further investigations on HR-related features of miRs profiles before and after NACT are needed.

In conclusion, our study revealed differences in miR-124a and miR-373 expression after NACT in primary BC tissues. Although miRs levels did not impact the response to NACT, we found miR-124a and miR-137 levels to be related to hormonal sensitivity of BC. Differential role of miRs in response to NACT with respect to hormonal receptors status needs further investigation.

## Data Availability

The datasets used and/or analysed during the current study are available from the corresponding author on reasonable request.

## Abbreviations

BC: Breast cancer
NACT: Neoadjuvant chemotherapy
HER2/neu: Human epidermal growth factor receptor 2
TN: Triple-negative
TNBC: Triple-negative breast cancer
HR: Hormone receptor
EMT: Epithelial to mesenchymal transition
OS: Overall survival
PFS: Progression-free survival
ER: Estrogen receptor
PR: Progesterone receptor
PCR: Polymerase chain reaction
ERRa: Estrogen-related receptor α

## References

[1] Siegel RL, Miller KD, Fuchs HE, Jemal A (2021). Cancer statistics, 2021. CA Cancer J Clin.

[2] Kurozumi S, Yamaguchi Y, Kurosumi M (2017). Recent trends in microRNA research into breast cancer with particular focus on the associations between microRNAs and intrinsic subtypes. J Hum Genet.

[3] Lu J, Getz G, Miska EA (2005). MicroRNA expression profiles classify human cancers. Nature.

[4] Izumiya M, Tsuchiya N, Okamoto K, Nakagama H (2011). Systematic exploration of cancer-associated microRNA through functional screening assays. Cancer Sci.

[5] Adi Harel S, Bossel Ben-Moshe N, Aylon Y (2015). Reactivation of epigenetically silenced miR-512 and miR-373 sensitizes lung cancer cells to cisplatin and restricts tumor growth. Cell Death Differ.

[6] Yang L, Song X, Zhu J (2017). Tumor suppressor microRNA-34a inhibits cell migration and invasion by targeting MMP-2/MMP-9/FNDC3B in esophageal squamous cell carcinoma. Int J Oncol.

[7] Lima CR, Gomes CC, Santos MF (2017). Role of microRNAs in endocrine cancer metastasis. Mol Cell Endocrinol.

[8] Iorio MV, Ferracin M, Liu CG (2005). MicroRNA gene expression deregulation in human breast cancer. Can Res.

[9] Ohzawa H, Miki A, Teratani T (2017). Usefulness of miRNA profiles for predicting pathological responses to neoadjuvant chemotherapy in patients with human epidermal growth factor receptor 2-positive breast cancer. Oncol Lett.

[10] Blenkiron C, Goldstein LD, Thorne NP (2007). MicroRNA expression profiling of human breast cancer identifies new markers of tumor subtype. Genome Biol.

[11] Alma DCP, Gerardo CM, Abraham PT (2017). Micro-RNAs as potential predictors of response to breast cancer systemic therapy: future clinical implications. Int J Mol Sci.

[12] Takahashi RU, Miyazaki H, Ochiya T (2015). The roles of microRNAs in breast cancer. Cancers.

[13] Kolacinska A, Morawiec J, Fendler W (2014). Association of microRNAs and pathologic response to preoperative chemotherapy in triple negative breast cancer: preliminary report. Mol Biol Rep.

[14] Negrini M, Nicoloso MS, Calin GA (2009). MicroRNAs and cancer–new paradigms in molecular oncology. Curr Opin Cell Biol.

[15] Kastl L, Brown I, Schofield AC (2012). miRNA-34a is associated with docetaxel resistance in human breast cancer cells. Breast Cancer Res Treat.

[16] Peurala H, Greco D, Heikkinen T (2011). MiR-34a expression has an effect for lower risk of metastasis and associates with expression patterns predicting clinical outcome in breast cancer. PLoS ONE.

[17] Eichelser C, Flesch-Janys D, Chang-Claude J (2013). Deregulated serum concentrations of circulating cell-free microRNAs miR-17, miR-34a, miR-155, and miR-373 in human breast cancer development and progression. Clin Chem.

[18] Tokumaru Y, Eriko K, Oshi M (2020). High expression of miR-34a associated with less aggressive cancer biology but not with survival in breast cancer. Int J Mol Sci.

[19] Chen F, Luo N, Hu Y (2018). MiR-137 suppresses triple-negative breast cancer stemness and tumorigenesis by perturbing BCL11A-DNMT1 Interaction. Cell Physiol Biochem.

[20] Lee SJ, Jeong JH, Kang SH (2019). MicroRNA-137 inhibits cancer progression by targeting Del-1 in triple-negative breast cancer cells. Int J Mol Sci.

[21] Ying X, Sun Y, He P (2017). MicroRNA-137 inhibits BMP7 to enhance the epithelial-mesenchymal transition of breast cancer cells. Oncotarget.

[22] Hafez MM, Hassan ZK, Zekri ARN (2012). MicroRNAs and metastasis-related gene expression in Egyptian breast cancer patients. Asian Pac J Cancer Prev.

[23] Kong W, He L, Richards EJ (2014). Upregulation of miRNA-155 promotes tumour angiogenesis by targeting VHL and is associated with poor prognosis and triple-negative breast cancer. Oncogene.

[24] Farsinejad S, Rahaie M, Alizadeh AM (2016). Expression of the circulating and the tissue microRNAs after surgery, chemotherapy, and radiotherapy in mice mammary tumor. Tumour Biol.

[25] Gasparini P, Lovat F, Fassan M (2014). Protective role of miR-155 in breast cancer through RAD51 targeting impairs homologous recombination after irradiation. Proc Natl Acad Sci USA.

[26] Huang Q, Gumireddy K, Schrier M (2008). The microRNAs miR-373 and miR-520c promote tumour invasion and metastasis. Nat Cell Biol.

[27] Jing SY, Jing SQ, Liu LL (2017). Down-expression of miR-373 predicts poor prognosis of glioma and could be a potential therapeutic target. Eur Rev Med Pharmacol Sci.

[28] Nakata K, Ohuchida K, Mizumoto K (2014). Micro RNA-373 is down-regulated in pancreatic cancer and inhibits cancer cell invasion. Ann Surg Oncol.

[29] Qu Y, Liu H, Zheng L (2017). Effects of microRNA-373 on the proliferation and invasiveness of breast carcinoma and its mechanisms. Zhonghua Yi Xue Za Zhi.

[30] Crosby ME, Kulshreshtha R, Ivan M, Glazer PM (2009). MicroRNA regulation of DNA repair gene expression in hypoxic stress. Can Res.

[31] Kofman AV, Kim J, Park SY (2013). microRNA-34a promotes DNA damage and mitotic catastrophe. Cell Cycle.

[32] Czochor JR, Sulkowski P, Glazer PM (2016). miR-155 overexpression promotes genomic instability by reducing high-fidelity polymerase delta expression and activating error-prone DSB repair. Mol Cancer Res.

[33] Ryspayeva D, Lyashenko A, Dosenko I (2020). Predictive factors of pathological response to neoadjuvant chemotherapy in patients with breast cancer. JBUON.

[34] Wang L, Asirvatham JR, Ma Y (2021). HER-2/neu-positive breast cancer neoadjuvant chemotherapy response after implementation of 2018 ASCO/CAP focused update. Breast J.

[35] Ogston KN, Miller ID, Payne S (2003). A new histological grading system to assess response of breast cancers to primary chemotherapy: prognostic significance and survival. Breast.

[36] Wei F, Cao C, Xu X, Wang J (2015). Diverse functions of miR-373 in cancer. J Transl Med.

[37] Wang LQ, Yu P, Li B (2018). miR-372 and miR-373 enhance the stemness of colorectal cancer cells by repressing differentiation signaling pathways. Mol Oncol.

[38] Zhou AD, Diao LT, Xu H (2012). β-Catenin/LEF1 transactivates the microRNA-371–373 cluster that modulates the Wnt/β-catenin-signaling pathway. Oncogene.

[39] Peng Y, Zhang X, Feng X (2017). The crosstalk between microRNAs and the Wnt/β-catenin signaling pathway in cancer. Oncotarget.

[40] Chen D, Dang BL, Huang JZ (2015). MiR-373 drives the epithelial-to-mesenchymal transition and metastasis via the miR-373-TXNIP-HIF1α-TWIST signaling axis in breast cancer. Oncotarget.

[41] Weng J, Zhang H, Wang C (2017). miR-373-3p targets DKK1 to promote EMT-induced metastasis via the Wnt/ β-catenin pathway in tongue squamous cell carcinoma. Biomed Res Int.

[42] Tessitore A, Cicciarelli G, Del Vecchio F (2014). MicroRNAs in the DNA damage/repair network and cancer. Int J Genomics.

[43] Han ZB, Yang Z, Chi Y (2013). MicroRNA-124 suppresses breast cancer cell growth and motility by targeting CD151. Cell Physiol Biochem.

[44] Chen Z, Liu S, Tian L (2015). miR-124 and miR-506 inhibit colorectal cancer progression by targeting DNMT3B and DNMT1. Oncotarget.

[45] Yang Q, Wan L, Xiao C (2017). Inhibition of LHX2 by miR-124 suppresses cellular migration and invasion in non-small cell lung cancer. Oncol Lett.

[46] Jiang CF, Li DM, Shi ZM (2016). Estrogen regulates miRNA expression: implication of estrogen receptor and miR-124/AKT2 in tumor growth and angiogenesis. Oncotarget.

[47] Zhou Z, Lv J, Wang J (2019). Role of microRNA-124 as a prognostic factor in multiple neoplasms: a meta-analysis. Dis Markers.

[48] Dong LL, Chen LM, Wang WM, Zhang LM (2015). Decreased expression of microRNA-124 is an independent unfavorable prognostic factor for patients with breast cancer. Diagn Pathol.

[49] Zhang L, Chen X, Liu B, Han J (2018). MicroRNA-124-3p directly targets PDCD6 to inhibit metastasis in breast cancer. Oncol Lett.

[50] Liu C, Xing H, Guo C (2019). MiR-124 reversed the doxorubicin resistance of breast cancer stem cells through STAT3/HIF-1 signaling pathways. Cell Cycle.

[51] Bi WP, Xia M, Wang XJ (2018). miR-137 suppresses proliferation, migration and invasion of colon cancer cell lines by targeting TCF4. Oncol Lett.

[52] Zhao Y, Li Y, Lou G (2012). MiR-137 targets estrogen-related receptor alpha and impairs the proliferative and migratory capacity of breast cancer cells. PLoS ONE.

[53] Onyido EK, Sweeney E, Nateri AS (2016). Wnt-signalling pathways and microRNAs network in carcinogenesis: experimental and bioinformatics approaches. Mol Cancer.

[54] Menck K, Heinrichs S, Wlochowitz D (2021). WNT11/ROR2 signaling is associated with tumor invasion and poor survival in breast cancer. J Exp Clin Cancer Res.

[55] Koni M, Pinnarò V, Brizzi MF (2020). The Wnt signalling pathway: a tailored target in cancer. Int J Mol Sci.

[56] Gonzalez DM, Medici D (2014). Signaling mechanisms of the epithelial-mesenchymal transition. Sci Signal.

[57] Caldon CE (2014). Estrogen signaling and the DNA damage response in hormone dependent breast cancers. Front Oncol.

[58] Jiménez-Salazar JE, Damian-Ferrara R, Arteaga M (2021). Non-Genomic actions of estrogens on the DNA repair pathways are associated with chemotherapy resistance in breast cancer. Front Oncol.

[59] Jansson MD, Lund AH (2012). MicroRNA and cancer. Mol Oncol.

